# Pathways and mixing of the north western river waters in the Black Sea

**DOI:** 10.1016/j.ecss.2020.106630

**Published:** 2020-05-05

**Authors:** S. Miladinova, A. Stips, D. Macias Moy, E. Garcia-Gorriz

**Affiliations:** Joint Research Centre, I-21027, Ispra, VA, TP 270, Italy

**Keywords:** Black Sea, Danube plume pathway, Mesoscale circulation, Rim current, Climatic changes

## Abstract

Given the increasing role of river-borne anthropogenically-derived substances on the water quality of impacted marine ecosystems, it is important to study the pathways of the river waters in the Black Sea. We perform tracer simulations for the river-borne substance, aiming to track its transport and accumulation in the basin and to identify possible long term trends. Our results suggest a decrease in Danube plume transport southward along the coastline and increasing transport to the north and north-eastern parts of the North Western Shelf (NWS) and then to the southwest. Over the 1960–2017 period, the Black Sea circulation showed an amplification and consolidation of the Rim Current, most likely in response to climatic changes. Recent changes in the circulation patterns seem to be a key factor for the modification of the river plume pathways. The concentration of the river-borne substance reaches an annual maximum in September, when pulses with larger tracer concentrations can be found in the inner basin. The accumulation of river-borne substance on the north and south shelf has increased in recent decades.

## Highlights

• Patterns of the north western river water pathways are explained.
• The Rim Current speed has been increasing for the past six decades.
• The maximum pulses of river water into the deep basin occur in September.
• Trends of river-borne substance accumulation over 1960–2017 are analysed.

## Graphical abstract

**Figure:**
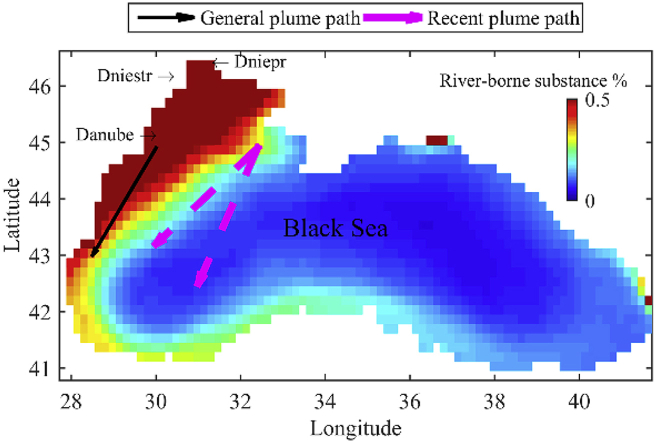

## 1 Introduction

**Fig. 1:**
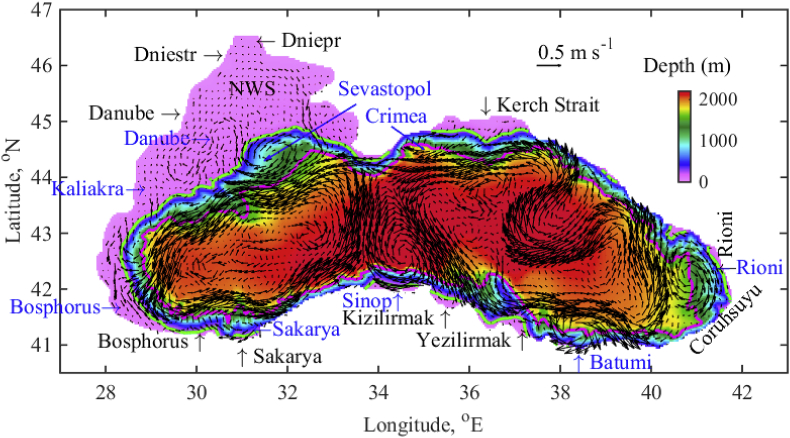
Bathymetry (colour bar) and map of the Black Sea. The 1500 m and 200 m isobaths are drawn in magenta and green. The names of the main rivers are written in black, while the names of several anticyclonic eddies are given in blue. An example of the Black Sea surface circulation, based on the simulated weekly mean velocity vectors and averaged over the upper 5 m depth and for the last week of August 2014, is represented by arrows, and the scale of 0.5 m s^−1^ arrow length is also given. (For interpretation of the references to colour in this figure legend, the reader is referred to the Web version of this article.)

The Black Sea is characterised by a positive water balance and is strongly influenced by the rivers' discharge. The NWS receives freshwater from major European rivers, including the Danube, Dniepr, and Dniester (Fig. 1). These big rivers, alongside some smaller ones, supply substantial amounts of bio-geo-chemical substances to the Black Sea (Panin and Jipa, 2002; Ragueneau et al., 2002; Lechner et al., 2014; Covaci et al., 2006). The environmental crisis and subsequent changes in the Black Sea's ecosystem and resources can be attributed to the natural and anthropogenic load of nutrients from the rivers (Yunev et al., 2007; Shalovenkov, 2000). The satellite data suggests a positive correlation between biological production on the NWS and along the Anatolian coast (Oguz et al., 2002), since the western shelf and coast, as well as the Anatolian shelf and coastal waters have high chlorophyll *a* concentrations almost continuously. The onshore and offshore meanders of the NWS plume support the phytoplankton production (Oguz et al., 2004). Realistic estimates of the micro plastic flow from rivers into the basin is an important factor for identification of plastic debris and for coordinating a response. The recognition of hazardous substances coming from rivers and their distribution and storage in the intermediate layers are of great interest for preserving the ecological integrity of the Black Sea (Humborg et al., 1997; Arvanitoyannis et al., 2008; Guieu and Martin, 2002). The understanding of Black Sea environmental dynamics remains incomplete as it is not known how nutrients and pollutants are transported between the coastal/shelf regions and open sea. Coordinating Black Sea protection measures requires a good understanding of the fate of river flow into the sea.

The spatial and temporal scales of river-borne substance spreading depend on the river discharge, the prevailing wind conditions (direction, speed and frequency), and the main sea current (the Rim Current) strength and pathway. All these factors display large seasonal and multi-annual fluctuations (Polonsky et al., 1997; Karageorgis et al., 2009; Malagó et al., 2017; Korotaev et al., 2003; Zatsepin et al., 2003; Kubryakov et al., 2018; Miladinova et al., 2017). In winter, the mixing is generally intense and deep, thus the river plume is not well visible. By spring time, the river plume flows on top of shelf water, which is itself strongly stratified from spring to autumn. Due to density stratification in the shelf, the surface and bottom water masses are distinct in their bio-geo-chemical properties. The mixing of the fresh and salt water on the shelf is restricted to the surface layers. The basic mechanism that controls the flow in the NWS is the spreading of the Danube outflow. During the transportation, the composition of the substances in the river water changes due to physical and biological processes. The substance distributions in the Black Sea upper layer appear to be sensitive to external pressures originating from regional weather variability (Konovalov and Murray, 2001; Oguz and Velikova, 2010) and anthropogenic inputs (Kıdeyş, 2002; Mee, 1992; Oguz, 2008). These distributions are influenced by the energetic eddy circulations, which are dominated by the Rim Current (Oguz et al., 1992; Özsoy and Ünlüata, 1997). Seasonal variability of the Black Sea eddies has been studied using hydrological data (Oguz et al., 1993), altimetry data (Korotaev et al., 2003; Kubryakov and Stanichny, 2015) and numerical simulations (Staneva et al., 2010; Stanev et al., 2002; Miladinova et al., 2017). The factors driving the basin seasonal variability are the disintegration of the Rim Current in late summer and the intensification of anticyclone activity. Typically in September, an abundance of anticyclonic eddies are formed between the shelf edge and the Rim Current, weakening the inner basin circulation.

The NWS freshwater plume usually forms a downstream current along the western and south-western coast, which is confined to the upper 25 m waters. The southward costal pathway is the most prominent because of the prevailing north-eastern (NE) wind over the basin, although other directions of the plume have also been reported (Karageorgis et al., 2009; Yankovsky et al., 2004; Kubryakov et al., 2018). When the plume is approaching the shelf break, the Rim Current cyclonic motion accelerates the freshwater stream. The Danube plume propagation during the summer period of 1992–2015, studied by Kubryakov et al. (2018) using of particle tracking model based on satellite altimetry measurements, wind reanalysis data, and chlorophyll *a* measurements (SeaWiFS, MODIS) found four different propagation pathways, namely: (i) In the years with prevailing NE winds, an alongshore southward current is formed along the NWS, near the western coast of the Black Sea. (ii) In the years with prevailing south-eastern (SE) winds, the mesoscale eddies effectively trap the Danube waters, transporting them to the deep western part of the basin; (iii) During several years, the Danube waters move to the east towards the western Crimean coast first and are then trapped by the Rim Current; (iv) A most unusual situation is observed during years with hot summers, when the north (N) winds bring the Danube plume to the north of the NWS, trapping it on the shelf.

There is however a lack of consistent data on the spatial spreading dynamics of river-borne substances through the shelf areas and their subsequent distribution in the open sea. In this paper we study the trajectory of the river waters through the Black Sea in order to further describe the spreading of substances coming from the rivers. All big Black Sea rivers (Fig. 1) are considered in this study, although particular attention is given to the rivers flowing into the NWS. A 3D hydrodynamic model (Miladinova et al., 2017) is linked to a passive tracer model to help visualisation of the river water pathway and address questions related to inter-annual and multi-decadal variability. Tracer simulations help to demonstrate the distribution of bio-geo-chemical substances coming from the rivers to the coast and shelf, shelf break and continental slope, and inner basin. We analyse the changes in the tracer pathways to the open sea in the last six decades (1960–2017) and identify trends.

## 2 Material and methods

**Fig. 2:**
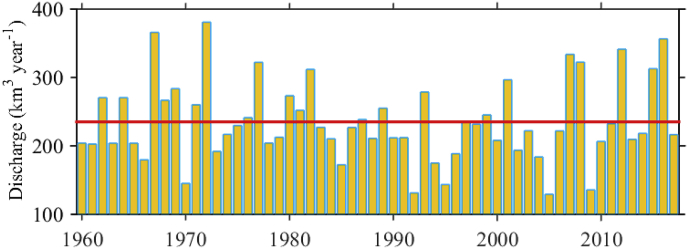
May–August mean Danube discharge (${\text{km}}^{3}{\text{ year}}^{-1}$). The red line marks the mean value 235 ${\text{km}}^{3}{\text{year}}^{-1}\;$ over the entire period. (For interpretation of the references to colour in this figure legend, the reader is referred to the Web version of this article.)

The Bosphorus Strait connects the Black Sea with the Mediterranean Sea via the Marmara Sea and the Kerch Strait is the connection with the Azov Sea (Fig. 1). The Black Sea shelf edge slope is steep and the shelf is narrow except for in the NWS region. The main Black Sea rivers considered herein are depicted in Fig. 1. A 3D hydrodynamic model has been developed as a basis for ecological modelling and long term Black Sea simulations (Miladinova et al., 2017, 2018). The model is based on a 3D hydrodynamic model comprising the 3D General Estuarine Transport Model (GETM, http://www.getm.eu/) and the General Ocean Turbulence Model (GOTM). The meteorological forcing from the European Centre for Medium Range Weather Forecast (ECMWF) available from http://www.ecmwf.int, based on 6-hourly records has been applied. It involves the ERA-40 project (1958–79) and ERA-Interim project (1980–2017). Monthly freshwater input has been estimated using the values from the Global Runoff Data Centre (GRDC, http://www.bafg.de/GRDC) runoff. The inflow of low salinity water (11‰) from the Kerch Strait (~50 ${\text{km}}^{3}{\text{ year}}^{-1}$), is considered in the model as the river runoff. According to the GRDC data set, the Danube has a mean annual discharge of 212, Dniepr - 55, Rioni - 14, Dniestr - 11, Sakarya – 7.3, Coruhsuyu - 6.7, Yesilirmak - 6 and Kizilirmak - 6 ${\text{km}}^{3}{\text{ year}}^{-1}$. For reference, the May–August mean Danube discharge in the period 1960–2017 is given in Fig. 2 and the mean value over the entire period is 235 ${\text{km}}^{3}{\text{ year}}^{-1}$ (red line). An example for the Black Sea surface circulation, based on simulated weekly mean velocity vectors, averaged for the upper 5 m depth and for the last week of August 2014 is given in Fig. 1 in order to illustrate the most energetic anticyclonic eddies, such as Sevastopol, Danube, Sinop, Battumi in the Black Sea.

**Fig. 3:**
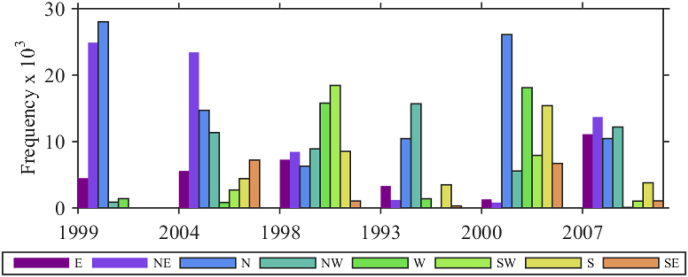
Monthly wind frequency for 8 directional bands, representing the probability of occurrence of winds with given direction and speed >7 ${\text{m s}}^{-1}$ in the NWS. The frequency is calculated from the atmospheric forcing wind speed and direction at 10 m height for the corresponding months and years in Fig. 4.

In order to identify the possible relationships between the plume propagation pattern and wind speed, direction and frequency, Fig. 3 shows the wind frequency for 8 directional bands, representing the probability of occurrence of winds in NWS with given direction and speed >7 ${\text{m s}}^{-1}$. The frequency is calculated from the atmospheric forcing wind speed and direction at 10 m height. The choice of individual years is made for the purpose of model validation against the particle tracking model by Kubryakov et al. (2018) and satellite optical data (MODIS and SeaWIFS). 1999 corresponds to the plume type (i), 2004 and 1998 – plume type (ii), 1993 and 2000 – plume type (iii), and 2007 – plume type (iv).

The passive tracer model was linked on-line via the Framework for Aquatic Biogeochemical Models (FABM, Bruggeman and Bolding, 2014) with the hydrodynamic model. Tracer experiments are carried out by solving the transport equation of a synthetic passive tracer. Details of the passive tracer equation and the assumptions made for the parameterization of turbulent fluxes (e.g. down-gradient fluxes with different diffusivities in vertical and horizontal direction) can be found in Burchard and Rennau (2008). The vertical diffusivity used within GETM is calculated within the GOTM model by solving a two-equation turbulence closure model based on the k-epsilon model. The tracer is loaded by the rivers, where its concentration is assumed to be 1, while the initial tracer concentration is 0 in the basin. Tracer initialisation starts in a beginning of each year and is then distributed through the basin during the year studied. The basin tracer concentration is reset to zero at the beginning of the first month of each year, and it represents yearly loading. The tracer is released uniformly across GETM vertical layers. Apart from the load from the rivers, the tracer distribution is expected to depend on the basin circulation. Since the mesoscale circulation is characterised by a class of energetic phenomena of time scales ranging from a few days to several months, we chose to analyse and discuss the weekly mean fields. A tracer concentration fraction (TCF) is introduced for detecting trends that are not influenced by the river discharge fluctuations. If the total amount of tracer released from all rivers until a particular month is *C*, the TCF of a grid box (x,y) with local tracer concentration c(x,y) and volume V(x,y), is defined as TCF=c(x,y).V(x,y)/C.

During summer stratification the thermocline is located at a depth of 20 m or less. A budget method is used to evaluate the coefficient of vertical diffusion (m^2^ s^−1^) in the upper halocline (20–50 m) of deep basin (with depth > 1500 m)

Kz=(∂c∂z)/(∫∂c∂tdz),

where c is the tracer concentration, z is the vertical coordinate (20–50 m), and t is time (from June 1st to October 31st). This approach assumes that time averaged advection in the basin is negligible. In closed basins during stagnant periods (June–October) this is regarded as a reliable assumption (Staalstrøm and Røed, 2016).

## 3 Results and discussion

### 3.1 Propagation of river outflow

**Fig. 4:**
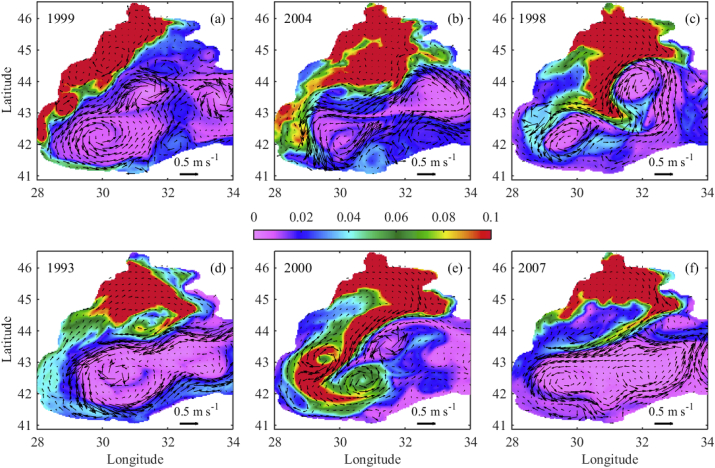
Weekly mean tracer distribution in the upper 5 m depth. (a) the first week of August 1999; (b) the fourth week of August 2004; (c) the first week of August 1998; (d) the third week of July 1993; (e) the fourth week of July 2000; (f) the second week of July 2007. The colour bar is in the interval [0, 0.1]. The speed (${\text{m s}}^{-1}$) and direction of the currents are given by arrows. The length of the 0.5 ${\text{m s}}^{-1}$ arrow sets the scale.

River outflows into the coastal ocean forms positively buoyant gravity currents. The properties of these fresher water currents are determined by the initial momentum at the river's mouth, as well as by interactions with coastal currents and winds. Under the influence of the Earth's rotation, this plume of fresher water tends to turn to the right in the northern hemisphere, flowing downstream as an alongshore current that stays trapped at the coast. Different types of NWS fresh water propagation patterns are captured in Fig. 4. We compare our results with those of the particle tracking model (Kubryakov et al., 2018) and satellite optical data (MODIS and SeaWIFS). The year 1999 corresponds to the plume type (i), 2004 and 1998 – plume type (ii), 1993 and 2000 – plume type (iii), and 2007 – plume type (iv). To facilitate reading, the tracer colour bar is set in the range 0–0.1. The tracer distribution visualises the pathway of river waters. The speed (${\text{m s}}^{-1}$) and direction of the currents are given by arrows, where the length of the 0.5 ${\text{m s}}^{-1}$ arrow is for scale. During the last week of August 1999 (Fig. 4a), the fresh water plume moves alongshore to the south, reaching the south-western coast of the basin. The plume includes several mesoscale anticyclonic eddies (MAEs), one of which is located just outside the Danube mouth, the so called Danube anticyclonic eddy (Korotaev et al., 2003). The other energetic eddy is located in front of Cape Kaliakra, in the narrowest part of the NWS (Fig. 1). A small anticyclonic eddy is present between the Danube and Kaliakra MAEs, which is called the Constanta eddy. The general path of the NWS fresh water flow, shown in Fig. 4a, agrees well with the literature (Fig. 5 in Kubryakov et al., 2018; Sorin et al., 2017). In this particular month the prevailing wind directions with speed above 7 ${\text{m s}}^{-1}$ are N and NE, and the May–August mean Danube discharge in 1999 is average (Fig. 2).

**Fig. 5:**
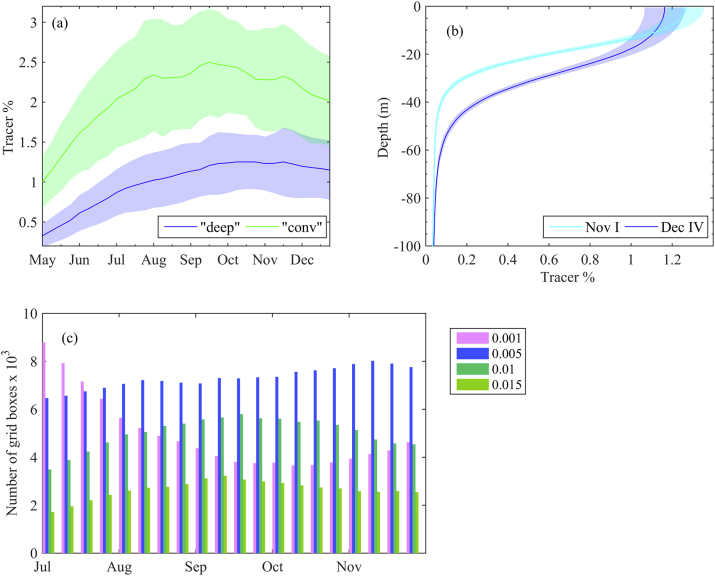
(a) Upper 10 m climatological mean tracer concentrations for the two specific zones: “deep” (depth>1500 m) and “conv” (200<depth≤ 1500 m); (b) Vertical tracer distribution in deep zone in the first week of November and the last week of December from the surface to 80 m depth; Shaded areas represent 95% confidence limits of mean. (c) Number of grid boxes in the deep zone with a specific tracer concentration within four tracer concentration bins (min-max): 0.-0.001; 0.001–0.005; 0.005–0.01; and 0.01–0.015. All values represent climatological mean averages over 1960–2017.

In the last week of August 2004 (Fig. 4b), the Danube plume turns to the north and then moves anticyclonically over the shelf to the south, where the Rim Current captures the plume and transports it to the south-west in cyclonic direction. The plume moves along the west coast to the south-west, as in the previous example, but is partially shifted eastward to the Rim Current in front of the Constanta eddy. Moreover, near the Bulgarian coast, the plume extended towards the end of the shelf break and MAEs emerged on both shelf and shelf break. Although NE and N winds prevail in August 2004, the river plume did not follow the usual path along the coast. It appears that a combination of intermediate and strong NE, N, and north-western (NW) winds (see bars for 2004 in Fig. 3) changed the typical path of the NWS waters. In 2004, the Danube discharge is below average (Fig. 2), therefore the southward transport along the west shelf is less intense. Our simulations are in good agreement with the satellite data for chlorophyll *a* (Fig. 8 in Kubryakov et al., 2018). Another type of NWS plume spreading is shown in Fig. 4c, which is characterised by similar flow of the Danube waters toward north and north-east part of the NWS like in Fig. 4b. The winds in July 1998 are favourable for shifting the plume eastward (western (W) and south-western (SW) winds prevail). At that time, the Rim Current is strong and close to the northern shelf-break, so it efficiently entrains the plume. The plume is inclined into the western gyre dividing it into two cyclonic eddies. The spreading of the NWS waters towards the western shelf is suppressed by the existence of MAE close to the Bulgarian coast. The year 1993 is characterised by a high Danube discharge and very low winter and annual mean sea surface temperatures (Miladinova et al., 2017). Additionally, the wind speed is low and N and NW winds prevail (Fig. 3). The Danube water flows north and north-east (Fig. 3d), as in the previous two examples for 2004 and 1998. An extensive anticyclonic eddy is evident in the deep part of the NWS (probably the Sevastopol eddy). Then, the Danube waters pass to the northern periphery of the Sevastopol anticyclone and are entrained in the orbital motion of the eddy. Further, the plume is transferred up to the west coast of Crimea. Interestingly, the plume in the third week of July 1993 is moving back to the NWS along the western coast, after reaching the Bosphorus.

During July 2000, the winds are strong and N, western (W) and south (S) winds are prevailing. The wind conditions are favourable for the north-east transport of the plume over the NWS. At the same time, the Rim Current in the west basin is disintegrated into two energetic cyclonic eddies (Fig. 4e). The presence of a strong mesoscale cyclonic eddy in the deep basin south of the north-western continental slope enhances the plume entrainment process, which causes the offshore spreading of plume waters. The plume is split in two branches due to a couple of energetic cyclonic and anticyclonic eddies in the south-western part of the deep basin. A part of the plume is spread into the basin and the rest is trapped by the MAE. This MAE induces plume spreading reversal currents that partially create a whirlpool at the NWS. Note that in July 2000 the western and south-western shelves are still unaffected by the NWS waters.

The last example (Fig. 4f) can be attributed to plume type (iv), as the Danube waters move northwards and then north eastward. An intense anticyclonic eddy, which geostrophic velocity reaches 0.4 m s^−1^, is generated on the NWS with 100–200 m depth. The eddy is located in the front of Constanta and close to the Rim Current. A less intensive MAE is located north-east of the previous eddy and they both direct plume displacement towards the north-east. Further up, with the help of another MAE (in the west of the Crimean Peninsula), the plume is directed to the Rim Current. It flows with the Rim Current until an intense MAE is reached, where the NWS waters are partially turned back into the NWS. The Bosphorus and other MAEs along the western coast constrain the NWS water spreading to the Anatolian coast. Tracer pattern in Fig. 4f is consistent with the MODIS chlorophyll *a* pattern on 15^th^ of July 2007. The May–August Danube discharge in 2007 is well above average and the strongest winds are eastern (E), NE, N and NW. In summary, the spatial patterns of NWS plume simulated here match well with the observed chlorophyll *a* patterns, illustrating the utility of the tracer model in identifying the NWS plume pathways. The above examples are insufficient to accurately classify the major pathways of river borne substances depending on wind conditions, May–August Danube discharge and inner basin eddy circulations. Meanwhile, on the base of the above examples we can distinguish three major pathways and the main assumptions are summarised in Table 1.

**Table 1:** Classification of the three major pathways depending on the wind direction and frequency, Danube discharges and the main cyclonic circulations.

Examples	Pathways	Prevailing winds	Danube discharge	Western basin cyclonic circulation
1999	(I) Southward along the western coast	N and NE winds	Average	Strong, disintegrated
2004, 1998 and 2000	(II) North and eastward, then southward, shifted eastward	Strong winds from different directions	Average or low	Average with two main eddies
1993 and 2007	(III) Trapped for a long period on the NWS	Low winds, stronger N and NW winds	High	Strong, integrated

### 3.2 Seasonal and inter-annual variability

Fig. 5a shows seasonal distribution of tracer concentration in the upper 10 m from May to November for two areas – “deep” (depth>1500 m) and “conv” (200<depth≤ 1500 m), as the mean of 1960–2017 seasonal patterns. The 95% confidence limits of the mean are also given. The tracer in the surface layer of deep waters reaches the first maximum in the second half of September. After a small decrease in October and beginning of November, the next maximum occurs in the second half of November. The tracer concentration in the deep basin is not more than 1.5% of the tracer concentration in rivers. Tracer distribution in “conv” exhibits three maxima – in the end of July, beginning of September and second half of November. The first maximum is related to the Danube discharge maximum, which typically occurs in May–July period. For the plume propagation type (I) and (II) (Table 1), the freshwater plume remains trapped in the NWS for a month or longer during summer. Furthermore, in the beginning of September the plume reaches “conv” and then “deep”, due to the MAEs intensification (the Sevastopol eddy, for example). The onset of vertical mixing typically takes place in late November, when the surface and intermediate layers mix. Therefore, the tracer surface concentration decreases due to deeper tracer penetration. For illustration, the climatological vertical tracer profiles in the first week of November and the last week of December are given in Fig. 5b. The vertical climatological tracer contours are averaged over the deep basin (with depth > 1500 m) and the 95% confidence limits of the mean are also given in Fig. 5b. Comparing both vertical profiles, it is obvious that the tracer decrease in the surface layer in December is due to its increase in the intermediate layers concentration.

For increased clarity when discussing the horizontal tracer spreading throughout the year, we consider four tracer concentration bins (min-max): 0.-0.001; 0.001–0.005; 0.005–0.01; and 0.01–0.015. We count the number of surface grid boxes from deep basin, in which the tracer concentration for a certain month is within a particular bin. The monthly mean number of grid boxes within particular bin is plotted in Fig. 5c. In the beginning of July, the number of boxes with high concentration (0.005–0.015) is small in comparison with the number of low concentration boxes. Then, the number of boxes with high tracer concentration begins to increase at the expense of the number of boxes with almost zero tracer concentration, and reaches a maximum in September, indicating that pulses of river waters into the deep basin typically occur in September. The number of boxes with intermediate tracer concentration stays at about 7x10^3^ from July to November. In November, the number of boxes with the lowest tracer concentration increases, showing the tracer vertical penetration in the subsurface layers.

**Fig. 6:**
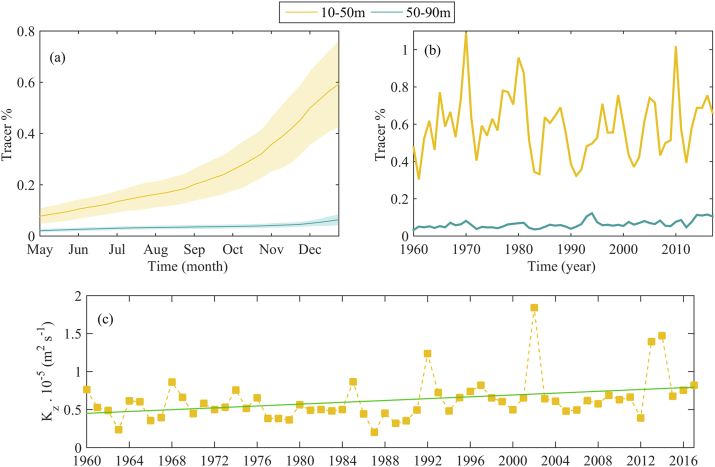
Climatological mean tracer concentrations in the deep zone (depth>1500 m): (a) Monthly mean of 10–50 m and 50–90 m layers (shaded areas represent 95% confidence limits of mean); and (b) Annual mean of 10–50 m and 50–90 m layers. (c) Annual mean vertical diffusion coefficient (x10^−5^ m^2^ s^−1^, presented by gold squares) and its trend line (drawn in green). (For interpretation of the references to colour in this figure legend, the reader is referred to the Web version of this article.)

Seasonal variations of the tracer concentration (%) in two intermediate layers (10–50 and 50–90 m) are given in Fig. 6a. The tracer concentration increases slightly until the beginning of autumn, when it begins to increase rapidly. By the end of August, the tracer concentration is about 0.2% of tracer concentration in the rivers and it reaches 0.6% by the end of December (Fig. 6a). While in the deeper layer 50–90 m, the tracer concentration increases slowly by the end of October, reaching 0.04% and then reaching about 0.06% by the end of December (not well visible in Fig. 6a). Thus, ten times less tracer is accumulated in 50–90 m than in 20–50 m. Comparing profiles presented in Fig. 6a with the “deep” profile in Fig. 5a, we can conclude that different mechanisms govern the tracer distribution in the surface and in the intermediate layers. In the surface, pulses of river-borne water flow into the inner basin and their further horizontal mixing due to mesoscale cyclonic eddies are key factors for tracer spreading in the inner basin. In the intermediate layers, the tracer distribution in spring - summer is mainly the result of anticyclonic activity, whereas in autumn – winter vertical mixing due to surface cooling begins to play a key role. The inter-annual variation of the mean tracer concentration in the intermediate layers is given in Fig. 6b. In the 20–50 m layer the tracer concentration is closely correlated to the Danube discharge (Fig. 2) with correlation coefficient R = 0.75 (0.6, 0.84), while there is no correlation between tracer concentration in the 50–90 m layer and the Danube discharge. So, the anticyclonic motion bringing the river water to the inner basin mostly affected the 20–50 m layer and its contribution on the deeper layer is negligible. No significant trend of tracer concentration in the 20–50 m layer is found, while there is a low increasing trend of the tracer concentration in the 50–90 m layer (0.007% per year).

Utilising the tracer spatial and temporal fields from June to August we estimate the vertical diffusion coefficient in the deep basin between 20 and 50 m depth (Fig. 6c). The long term (1960–2017) averaged vertical diffusion coefficient is 7.1x10^−6^ (±8.4x10^−7^) m^2^ s^−1^, which is in accordance with the results in Samodurov and Ivanov (1998). Additionally, we establish a small positive trend of vertical diffusion coefficient in the upper pycnocline (10^−7^ m^2^ s^−1^ per year). The possible reason for this trend is the cold intermediate layer distortion in recent years (Miladinova et al., 2017, 2018). Both increasing trends, namely tracer concentration in the 50–90 m layer and that of vertical diffusion coefficient, confirm our hypothesis that vertical mixing in autumn-winter is the main mechanism of tracer spreading into the 50–90 m layer.

### 3.3 Trends

**Fig. 7:**
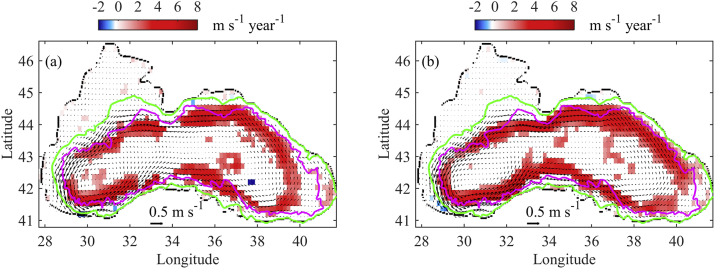
Climatological surface velocities (${\text{m s}}^{-1}$, arrows) and speed trends (${\text{m s}}^{-1}\text{ per}$ year x 10^−3^, colour bar) with *p-value* < 0.05 over 1960–2017 (a) in September; (b) in November. The grid boxes without any significant trend are displayed with white colour. Surface velocities are 30 m vertical mean values. The 1500 m isobath is drawn in magenta and the 200 m in green. (For interpretation of the references to colour in this figure legend, the reader is referred to the Web version of this article.)

For the purposes of trend analysis, the tracer and velocity fields are interpolated on a coarser grid (10 × 10 min). This interpolation is necessary due to the large volume of simulation results to be processed. All trends are calculated for the entire simulation period (1960–2017). Trends of system circulation for September and November are given in Fig. 7. We hypothesise the strengthening of the basin scale cyclonic circulation and the intensification of mesoscale activity (Miladinova et al., 2017). Climatological horizontal velocities are calculated in each grid box of the coarse grid and averaged over the upper 30 m. For the grid points in the NWS with depth less than 30 m, the velocities are averaged over the current depth. The climatological velocities and their directions are represented by arrows. The speed variation (${\text{m s}}^{-1}\text{ per}$ year x 10^−3^) at each grid point is calculated and depicted in Fig. 7 by different colours (blue – decrease, white – no change or no significant trend, and red – increase). Only grid boxes with statistically significant trends (*p-values*<0.05) are depicted in Fig. 7. It is seen that in September plenty of MAEs appear between the shelf edge and the Rim Current and persist in subsequent months (Oguz et al., 1993; Korotaev et al., 2003; Staneva et al., 2002), while the Rim Current is disintegrated in the rest of the basin. Since the MAEs are not permanent, they are not apparent in the climatological flow field (Fig. 7). However, the comparison between climatic fields in September and November shows similarity in terms of speed and integrity of the Rim Current. For example, the maximum mean current speed of the upper layer (30 m depth) is about 0.43 m s^−1^ for both months. A significant increasing trend is found for the Rim Current speed with the maximum increase of about 0.006 m s^−1^ per year in the north-eastern and south-western part of the Rim Current. The Rim Current speed increases drastically in the vicinity of the Crimea Peninsula and along the Anatolian coast. Minor trends are established for the inner parts of the western and eastern basins. Consequently, increased current speed must be largely responsible for the changes in the NWS plume spreading, as the Rim Current is a key factor for the plume spreading. The difference between the September and November trends lies in the larger area of speed increase in November. In particular, the Rim Current speed increase covers the easternmost part of the basin. Note that the increasing trend in both month covers mostly the Rim Current path in the deep basin and there in no significant trend on the NWS and shelf break (magenta line denotes the 1500 m isobath in Fig. 7).

**Fig. 8:**
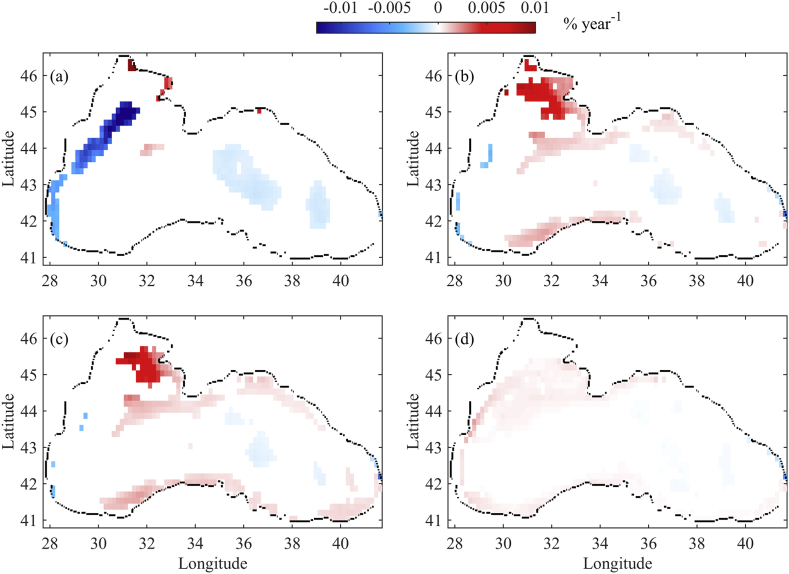
Trends of TCF (% per year) in September with *p-value* < 0.05 over 1960–2017. (a) In the upper 10 m; (b) in 10–20 m; (c) in 20–30 m; and (d) in 30–40 m.

To explore the changes in plume distribution over the simulation period, we choose to present the TCF linear trends in September. TCF in different water layers is calculated and interpolated on the coarser mesh. Linear trends are then sought, just as for the upper basin circulation, and TCF percentage change per year is plotted in Fig. 8. The colour bar is fixed in the interval [-0.01 0.01], where the blue colour represents the negative values, white – zeros or no significant trend, and red – positive values for all plots. In the surface layer (0–10 m) a strong decreasing trend (−1.5x10^−2^% year^−1^) is established on the NWS in the area with a depth between 60 and 200 m over the simulation period. A weaker decreasing trend is identified along the Western Shelf (Fig. 8a). The decreasing TCF trends are a result of the NWS plume pathway changes on the surface. This is due to the north and north-western path of the Danube plume on the NWS (Fig. 4c–f) and the Rim Current speed increase in the inner deep basin (Fig. 7a). Instead of flowing closely along the western coast, the plume moves initially north and north-east. The Rim Current captures the NWS plume on the northern shelf break and moves it south without mixing it with the western coastal and shelf waters.

In the inner part of the central and eastern part of the basin, a low decreasing trend in TFC is found. TCF trends in the layers 10–20 m and 20–30 m are quite similar (Fig. 8b and c). In the shelf areas, once again, the largest changes are found. The tracer tends to increase in north and north-eastern regions by a maximum percentage of ~9x10^−5^% year^−1^. The TCF increase is again attributed to a change in the Danube water pathway. The Danube plume moves towards the north and north-eastern part of the NWS more frequently than directly to the south. The river waters remain locked in the north and north-eastern part of the NWS up to a month or longer, where the river-borne tracer is horizontally and vertically mixed. The lack of a significant increase in the TCF trend on the surface is probably associated with the greater variation in surface properties, even in the presence of a slight wind curl. Increasing trends of TCF are established in the outer periphery of the Rim Current as a result of the Rim Current acceleration that prevents horizontal mixing of the surface waters. While in the upper 30 m significant trends are established for the shelf zones, trends in the 30–40 m layer are very weak (Fig. 7d).

As previously stated, three major river plume pathways are suggested. Based on trend analysis of the passive tracer coming from the rivers, a decrease of the tracer concentration is found along the typical pathway (I). One possible explanation for this somewhat unexpected trend in tracer concentration is that the river plume favours other pathways (II) and (III). It seems that the changes in the local weather conditions (e.g. winds and precipitations) and their effect on the river discharges and basin circulations are the key factors for the variation of the plume pathway. Thus, an increasing accumulation of river-borne hazardous substances, micro plastic or other matter is expected on the north and north-eastern part of the NWS.

## 4 Conclusions

The results presented herein show the complex hydrodynamic conditions that control the evolution of the NWS rivers’ plumes and the transport of the river-borne materials in the open sea. The Black Sea exhibits a unique environment, where complex shelf and shelf break topography, variable wind forcing and strong system current actively impact the dynamics and the transport of the freshwater plume. The present study makes use of tracer model simulations to highlight and analyse the pathways and mixing of riverine waters. Our results for the river water pathways are in agreement with previous studies and satellite images of chlorophyll *a*.

Evaluation of the various pathway patterns in relation with the winds direction and frequency, the Danube discharge and system current suggests three major pathways for the river-borne substances. (I) The southward pathway along the western coast in the case of prevailing N and NE winds, average Danube discharge and strong but disintegrated main current; (II) Initially directed to the north and east, then turning to the south and being shifted eastward in the case of strong winds from different directions, average or low Danube discharge and average Rim Current; (III) Trapped for a long period on the NWS, then turning to the south and being shifted eastward in the case of strong N and NW winds, high Danube discharge. The typical pathway (I) has recently appeared to be less preferential, leading to an increasing accumulation of the river-borne substances in the north and north-eastern part of the NWS. It is likely that over the 1960–2017 period, the Black Sea circulation has experienced changes and the intensification of the Rim Current speed in summer – autumn is one of them. Our results show that the river-borne substances achieve maximum concentrations in the surface of the inner basin in September. Moreover, in September, the strongest offshore pulses of river waters are estimated.

Although our simulations are focussed to the study area, similar scenarios can be envisioned in other estuarine or semi-enclosed systems characterised by strong river inflow impact, such as the semi-enclosed Mediterranean regions: North Adriatic Sea and North-Western Aegean Sea (Kourafalou, 2001; Kontoyiannis et al., 2003) or the Baltic Sea (Radtke et al., 2012; Soosaar et al., 2016). Tracer simulations help to identify the river plume pathways and to assess the impact of the freshwater discharge on stratification and circulation in coastal, shelf and open sea areas, as well as on transport and fate of river-borne matter (nutrients, anthropogenic pollutants, litter). Moreover, as the freshwater fluxes represent a major link between the terrestrial and marine areas, they can be considered as a key factor in the evolutions of the coastal ecosystems which closely depend upon the river inputs.

## CRediT authorship contribution statement

**S. Miladinova:** Conceptualization, Methodology, Software, Writing - original draft. **A. Stips:** Supervision, Project administration, Funding acquisition, Writing - review & editing. **D. Macias Moy:** Conceptualization, Methodology, Writing - review & editing. **E. Garcia-Gorriz:** Data curation, Visualization, Software.

## Declaration of competing interest

The authors declare that they have no known competing financial interests or personal relationships that could have appeared to influence the work reported in this paper.
